# The Metaverse, Religious Practice and Wellbeing: A Narrative Review

**DOI:** 10.1089/cyber.2023.0003

**Published:** 2024-01-08

**Authors:** Justin Thomas, Mohammad Amin Kuhail, Fahad AlBeyahi

**Affiliations:** ^1^ King Abdulaziz Center for World Culture Dhahran (Ithra/Sync), Dhahran, Saudi Arabia.; ^2^ College of Interdisciplinary Studies, Zayed University, Abu Dhabi, United Arab Emirates.

**Keywords:** metaverse, religion, Internet, wellbeing, psychopathology

## Abstract

The metaverse is touted as the next phase in the evolution of the Internet. This emerging digital ecosystem is widely conceptualized as a persistent matrix of interconnected multiuser, massively scaled online environments optimally experienced through immersive digital technologies such as virtual reality (VR). Much of the prognostication about the social implications of the metaverse center on secular activities. For example, retail, entertainment (gaming/concerts), and social networking. Little attention has been given to how the metaverse might impact religion. This narrative review explores contemporary research into online religious practice and the use of immersive digital technologies for religious purposes. This focus informs a discussion about how the metaverse, an online and immersive technology, might impact religion/religious practices. For billions worldwide, religion is an essential aspect of social identity and a cornerstone of psychological wellbeing. The emergence of the metaverse may represent a new way of connecting with an ancient source of human flourishing.

## From Cave Walls to Paywalls

The appearance of cave art during the upper paleolithic (50–10 thousand years ago) is linked with the emergence of cognitively modern humans. Burns,^1^ for example, argues that the appearance of cave art is evidence of complex brain reorganization in anatomically modern human beings. Similarly, with the same time frame in mind, Nakazawa^2^ posits the development of a “fluid mind”: a new cognitive configuration capable of unprecedented acts of creativity yet, at the same time, a mind prone to delusion. Nakazawa writes:

Only *Homo sapiens* has obtained a fluid mind independent of the external world. Thus, we can even describe the modern human being as a kind of “mad” animal for the ability to conceive things that do not exist. (p. 36)

This human capacity and desire to project our inner worlds outwardly led to the creation/recreation of hunt scenes on cave walls. Occasionally, such cave art also depicts imaginary beings—for example, therianthropes (part human, part animal)—things we probably never saw in the physical world.^3^ This human urge to recreate our physical environments or project unseen inner worlds is currently manifesting in an idea known as the metaverse.

Conceptually, the metaverse promises to offer a computer-rendered copy of the physical world along with much of its contents. In the language of the metaverse, such replicas of the physical world and its contents (people and things) are called digital twins.^4^ Beyond these pixel-based rerenderings of our physical (atom-based) reality, the metaverse will also offer artefacts, environments, and beings we never saw before. However, unlike the static, two-dimensional (2D) renderings/drawings of our Paleolithic ancestors, the architects of the metaverse (technology companies) have higher aspirations. The current vision is to create immersive, three-dimensional (3D), interactive experiences akin to a parallel plane of existence: a digital ecosystem populated by billions, sitting atop our physical world, fully integrated with the global economy.^5^

## The Metaverse

The concept of parallel worlds can be traced back to ancient mythology and features prominently in early articles of science fiction.^6^ The term metaverse, however, first appears in the Neal Stephenson novel *Snow Crash*.^7^ Stephenson uses the term to describe a persistent virtual world, a successor to the Internet, populated by millions of people in digital avatar form. This fictional virtual reality (VR) features places of work (offices), rest (homes), and play (nightclubs), where people meaningfully interact with each other across an expansive digital ecosystem.^8^

This fictional envisioning of a metaverse resembles its emerging factual counterpart. This embryonic idea, the metaverse, is heralded as the 3D web, Web3 (not to be confused with Web 3.0) and the next phase in the evolution of the Internet.^5^ Also aligned with Stephenson's vision, the proposed use of immersive technologies, such as virtual and augmented reality, will result in interconnected virtual environments where people and computer-generated characters, in avatar form, purposefully interact.

Conceptually, the metaverse is still unfolding. There is no universally agreed definition; even the term metaverse remains contested. At present, numerous definitions intersect and overlap, providing at least the semblance of where our relationship with technology is heading. For example, Ball defines the metaverse as:

A massively scaled and interoperable network of real-time rendered 3d virtual worlds that can be experienced synchronously and persistently by an effectively unlimited number of users with an individual sense of presence and with continuity of data such as identity, history, entitlements, objects, communications, and payments. (p. 576)

With more of an emphasis on function, the technology company Meta (formerly Facebook) describes the metaverse as:

… the next evolution in social connection and the successor to the mobile internet … a set of digital spaces that you can move seamlessly between. Like the internet, the metaverse will help you connect with people when you aren't physically in the same place and get us even closer to that feeling of being together in person.^9(p1)^

Although the metaverse remains conceptually inchoate, several commentators agree that games such as Second Life, Fortnite, Minecraft, and Roblox represent “proto-metaverses.” They each reflect at least some components of the envisioned metaverse.^10,11^ These proto-metaverses can be viewed as the direct antecedents of the metaverse-proper, potentially offering us a glimpse into the metaverse's functioning and likely socioeconomic impact.^12^

Second life, for example, Linden Lab's multimedia platform launched in 2003—encourages users to create and control avatars, which socially interact within a virtual world. This proto-metaverse is a persistent and seamless online environment (a digital world) where individuals can roam around without necessarily having a predetermined objective. Additionally, much game content is user generated and user owned, opening the door to economic opportunities, such as buying and selling.^13^ At peak popularity, Second Life attracted real-world, for-profit, and nonprofit organizations (Adidas, BBC, Save the Children) keen to establish a presence inside the virtual world. This type of engagement went beyond mere advertising and brand placement. For example, Harvard University's law school offered complete courses that could only be taken within the platform.^5^

Roblox, another proto-metaverse, has enjoyed massive popularity in a very short period. Roblox is both an online game platform and a game creation system. If second life is a virtual world, then Roblox is a virtual galaxy, allowing its users to visit and create many different virtual worlds. Similarly, Minecraft also allows users to create new worlds. Roblox and Minecraft are sandbox games; they allow their users (currently 150–300 million) to shape the gameplay environments.^14^ Since 2016, both Roblox and Minecraft have allowed users to engage with more immersive versions of their offerings through VR headsets.^15^

A significant limitation of these technologies—why they are not metaverses—is their current lack of interoperability. For example, my Roblox avatar cannot wander across to Second Life, and I cannot spend my Minecoins (Minecraft's in-game currency) in the Fortnite item shop. Furthermore, the fully-fledged metaverse is envisaged as going beyond recreation (gaming), offering environments for retail, commerce, education, health care, and more.

The metaverse will go beyond gaming. However, it will be experienced through the immersive digital technologies (VR) we typically associate with 3D gaming.^11^ The current discourse about the metaverse emphasizes the realism and the social aspects of the experience. VR (and other immersive technologies) provides a sense of *presence*: the perceptual illusion of nonmediation.^16^ Additionally, the multiuser nature of the metaverse affords *social presence*: the illusion that we are inhabiting the virtual environment with others.^17^ The metaverse, then, is a place where individuals will embody 3D avatars—our digital twins through immersive technologies—and interact in meaningful ways with other humans and artificially intelligent agents. All these components, 3D-rendered environments, the Internet and VR, already exist. However, the convergence of existing technologies can often be greater than the sum of the parts. Such a convergence (the metaverse) has the potential to bring about catalytic social change, transforming many aspects of our lives.

While the full implications of this emerging online ecosystem are unknown, some authors, somewhat hyperbolically, describe it as the “digital big bang.” However, rather than being analogous to new beginnings, the metaverse (the Internet) has also been viewed as a harbinger of the end, a digital forerunner to the parousia (the second coming of Christ). Within various eschatological traditions, the Internet, and by extension, the metaverse, has frequently been interpreted as a sign of the end of time.^18^ Whether viewed positively or negatively, the implication is that the metaverse, will usher in a new epoch for humanity.^11^ Somewhat less dramatically, however, Ball^5^ suggests the magnitude of transition will be akin to going from the fixed-line (dialup) Internet of the 90s (Web 1.0) to the contemporary era of 5G mobile Internet and cloud computing (Web 2.0). The transition between Web 1.0 and 2.0 popularized the phrase “disruptive innovation,” the process whereby new ways of doing things displace conventional products and services. The transition from Web 2.0 to the metaverse will, at the very least, have no lesser social impact.

Speculation about the nature of this impact has emphasized a shift toward realistic real-time collaboration and social engagement. Beyond enhancing the ability to communicate globally, the metaverse promises environments where global, real-time, virtual collaboration is possible and preferable to its physical and web2.0 counterparts. Commenting on the metaverse's potential for social good, Duan et al.^19^ suggest that the transition to virtual environments has the potential to weaken the concepts of race, gender, and even physical disability. However, such a development's inherent “social good” is contestable. Is masking race, gender, and disability behind an avatar inherently beneficial to society? Would the “weakening” of such concepts ultimately lead to the sociocultural homogenization of our online world? And how might that be good for society?

Although the benefits of greater online collaboration and social engagement are far from clear-cut, this does appear to be a likely direction of travel. For instance, this migration to online social spaces came to the fore, through existing technologies (proto-metaverses), during the COVID-19 pandemic.

For example, infection control measures meant that, in some cases, events such as graduation ceremonies and live music concerts were conducted in virtual worlds like Minecraft^20^ and Fortnite.^5^ A fully fledged metaverse might eventually lead to the preferencing of the virtual over the physical in many social and occupational domains. Virtual events are certainly lower cost and carry a lower risk of physical harm.

## Religion

Forecasting the social impact of the metaverse tends to center on secular activities such as retail, entertainment, and tourism. However, more attention should be given to the metaverse's implications for religion and religious practice. Whether one believes in the truth of religion or not, its widespread influence on human behavior is undeniable and significant. For around 84 percent of the world's population, religion impacts important psychological constructs such as motivation, values, self-concepts, morality, and more.^21^ Unsurprisingly, with such a matrix of psychological implications, religion also plays a critical role in emotional wellbeing, resilience, and mental health.

Religion, or religiosity (enacting religion), can give people a strong sense of belonging to a valued social group (positive social identity). This social identity in and of itself positively impacts health and wellbeing (for review, see Haslam et al.^22^). In addition to providing many people with a valued social identity, social connection, and a sense of belonging, religiosity is also characterized by a shared creed that typically elicits commitment to a set of advocated attitudes and practices, for example, scriptural reading, meditation, prayer, and fasting.^23^ Furthermore, an expansive body of research suggests that religiosity is associated with better mental health. In particular lower rates of depression^24–27^ and substance-related disorders.^28^ For example, a meta-analytic review spanning 147 studies (pooled sample of 98,975 participants) confirms the negative correlation between religiosity and depression.^29^ Similarly, a prospective longitudinal study following participants over a decade also supports the idea that religiosity reduces the risk of depression, especially among those deemed “high-risk” from a genetic standpoint.^23^

Similarly, A U.S.-based epidemiological study integrated data from three large cohorts (*N* = 92,008). The study reported that participation in religious services was associated with greater longevity, better mental health, and greater psychosocial wellbeing, even after controlling many other potential confounding variables.^26^ Such findings are widely replicated beyond Judeo-Christian and North American contexts. For example, similar findings are reported for Muslims in the Arab world (for review, see Thomas^30^).

Attempting to identify possible causal mechanisms underlying the association between religion and mental health, Pargament^31,32^ proposed the idea of positive religious coping. This idea was grounded in the observation that people's cognitive and behavioral responses to stressful life events are frequently informed by their faith traditions. The religious coping hypothesis suggests that adaptive faith-informed strategies, such as prayer, focusing on the afterlife, and seeking support from clergy, can help prevent the onset of mental health problems. Pargament argues that such adaptive (positive) religious coping strategies reflect beliefs about the meaningfulness of life and a reliance on a secure relationship with a merciful God.^32^ A growing body of evidence, cross-sectional and prospective longitudinal studies, offer support for this mental health-promoting/preserving function of religious coping.^33–35^ For example, in one longitudinal study among adult members of a protestant church, positive religious coping appeared to buffer the deleterious effects of adverse life events on depression even after controlling for other potentially confounding variables.^36^

How the metaverse might impact or interact with religious practices remains an open question. Furthermore, given the observed public health benefits associated with religious practice,^26,37^ this question is undoubtedly an important one.

## From Metaverse to Metachurch

The metaverse will undoubtedly have implications for religion. For example, most of the world's major religious traditions emphasize congregational (social) and interpersonal practices. One of the main attributes of the envisioned metaverse is social presence, a sense of being together with other people. This concept lends itself well to congregational activities. Meta (formerly Facebook), a pioneer in metaverse technology, articulates its vision of the metaverse as a technology that can “… get us even closer to that feeling of being together in person.”^9(p1)^ The aspiration to expand into the religious realm is explicit; the company recently appointed a global director of global faith partnerships.

Similarly, speaking at the 2021 Virtual Faith Summit, Meta's Chief Operating Officer said: “Our hope is that one day people will host religious services in virtual reality spaces as well, or use augmented reality as an educational tool to teach their children the story of their faith.”^38^ There are also religious organizations, such as Life.Church that place emerging technology at the center of their work. At present, Life.Church Online offers over 90 services per week across 5 different digital platforms. Another recent Life.Church development is their Bible Lens application, which uses artificial intelligence (machine vision) to suggest biblical verses related to photo-like content.^39^ Similarly, with VR at its core, there is also an organization, VR Church, describing itself as: “a spiritual community that exists entirely in the metaverse to celebrate God's love for the world.”^40(p1)^ The VR Church, its Web site informs us, uses immersive VR to hold weekly services, where congregants gather in avatar form for prayer meetings, bible studies, and other social events.

An earlier, well-documented attempt at establishing a virtual church was the 2004 research project known as the Church of Fools (CoF).^41^ Supported by the Methodist Church of England, the project involved virtual worshipers (up to 30 at a time) attending services at a 3D virtual church. Congregants would embody cartoon avatars, while prominent ministers in avatar (“revatar”) form would lead services. The project was intended as a 3-month investigation into whether Internet-savvy Christians and (non-Christians) might embrace a VR church. The answer was an emphatic yes, and at the end of the project, the congregants were unwilling to let the community die and continued meeting, although through a 2D chatroom. In evaluating the CoF, Kluver and Chen^41^ suggest that the project “fused a material sense of spirituality with the virtual world created online” (p. 117). The CoF also attracted a new demographic into the church in the form of relatively young (under 30) males.

Beyond virtual congregations, the idea of using digital technologies, particularly VR, has spread to other religious practices. For example, using VR to facilitate cyber pilgrimages has gained growing interest recently.

Hill-Smith^42^ suggests that beyond idle curiosity and entertainment, the cyberpilgrimage can be a helpful way of preparing, psychologically and informationally, for an actual (terrestrial) journey to a pilgrimage site. Although cyberpilgrimage raises issues of experiential authenticity, Hill-Smith argues that these digitally mediated online experiences can be intensely charged, transformative, enlightening, and profoundly fulfilling on both emotional and spiritual levels. In 2021, King Abdul Aziz Center for World Culture, based in Saudi Arabia, made available a VR tour of several of Islam's holiest sites, including Masjid al-Haram in Mecca (The Kaaba) and the Prophet's Mosque in Medina.^43^ Physically visiting such places is restricted to Muslims, while VR visits are open to all. This accessibility is another example of how religion online and, eventually, the metaverse can transcend current demographic norms for religious participation.

Setting aside 3D digital environments and VR, many religious institutions and individual practitioners regularly use the Internet to facilitate faith-based practices. For example, findings from the Pew Internet and America Life project reported that two-thirds of adult Internet users have engaged in online faith-related activities.^44^ Surprisingly, however, very few studies have yet to explore the extent that online participation in religion is associated with the same wellbeing benefits reported for in-person religious practice.^45^ One study that attempted to explore this question used a cross-sectional survey design, spanning practitioners of the world's five major religious traditions: Judaism, Christianity, Islam, Hinduism, and Buddhism.^46^ The study confirmed that many psychological and social benefits derived from in-person participation in religious practices also accrue for those participating in virtual forums. Furthermore, the study's authors reported that online forums were more accessible, attracting the unaffiliated, and those already actively engaged in their local religious organizations.

However, the dearth of research in this area must be addressed before we conclude that online religious participation confers similar wellbeing benefits to those observed from its in-person equivalent.

## COVID-19: Exodus to the Web

Historically, religious institutions have used online platforms to supplement and complement their in-person services. During the pandemic, however, online activity became the primary medium of engagement.^47^ In many places, stringent infection control measures, such as social distancing protocols, brought the congregational religious practice into the online sphere. Despite pockets of public protest and resistance, information communication technologies enabled the continuity of congregational religious practices. This situation presented a further opportunity to explore the acceptability and impact of digitally mediated religious participation.

A study exploring longitudinal data for around nine thousand U.K. adults looked at the association between the frequency of online religious participation during lockdown (March 23 to May 13, 2020) and various indicators of psychological wellbeing and mental health.^45^ The findings suggest that those who participated in online religious practice (for at least 1 week) had higher levels of life satisfaction and subjective wellbeing than their nonparticipating peers. Moreover, these differences remained even after the data were adjusted for baseline sociodemographic characteristics and prepandemic levels of in-person religious participation.

Another study during the pandemic looked at factors predicting the acceptance and eventual transition to online religious practice. Among other factors, the best predictor of actual participation in online congregations was attitudinal, specifically, the view that digitally mediated congregations are a viable medium for fulfilling spiritual goals.^48^ The authors, however, note that during the online religious services in their study, peer-to-peer social interaction was absent, making the online congregation more of a useful workaround than an authentic and socially fulfilling religious activity.

While the pandemic necessitated digital alternatives, it is essential to note that not all religious denominations presently sanction the performance of all religious practices online. For example, in 2005, at the pontifical council for social communication, the Roman Catholic Church declared that the sacraments (e.g., communion, confession, and last rights) could not be validly performed online.^49^ During the COVID-19 pandemic, proposals to undertake the sacrament of confession online (through live stream) were blocked. The objection was based on a requirement for the priest and penitent to be in the same physical space.^50^ Pope Francis urged the faithful to confess directly to God, with the resolve of later performing the sacrament in the traditional person-to-priest manner when safe to do so.^49^ Even when they are sanctioned for online performance, religious practices are viewed as lacking authenticity, being less valid, and perhaps less spiritually enriching than their in-person counterparts. For example, Kluver and Cheong^51^ interviewed religious leaders from among Singapore's Christian, Buddhist, Hindu, and Muslim faiths.

They found nearly unanimous agreement across traditions that the Internet was not an acceptable substitute for offline religious participation. The objections to “online worship” revolved primarily around two key arguments. The first is that worship is intensely interpersonal, and the artificiality and impersonal aspect of the Internet does not bring one into contact with God.

Whether the metaverse, with its aspirations of ecological realism (digital twinhood), presence, and social presence, will help assuage these criticisms remains to be seen. However, when considering whether the digital space can function as a “sacred space,” Kulver and Chen^41^ argue that historically the technology involved in facilitating worship typically transcends its function role:

Gothic arches were designed to inspire awe, and thus invite reflection upon God, and stained-glass began as an attempt to teach largely illiterate peoples basic stories of the Bible. In both of these cases, the technological basis became more sophisticated, and came to be seen as beautiful, irrespective of their religious intention. In both cases, also, the relationships enacted within the technology, relationships with other humans and with God, eventually transcended the technologies themselves. (p. 138)

Could the virtual places of worship on the metaverse, however they are designed, similarly transcend the technology? Would this allow the metaverse to offer “sacred spaces,” sacred at least in the estimation of those who worship there? Put another way, is there a digital equivalent to consecrated ground, and who consecrates it?

Such questions raise vital issues in the discussion about the limitations of online offerings. Beyond the realism that XR and 3D-rendered virtual environments afford, the issue of legitimacy rests with senior religious leaders and figureheads. As previously discussed, the Vatican's current position is that the sacraments of the Catholic Church cannot be validly performed online.^49^ Within the Sunni Islamic tradition, however, authority is less centralized, and acts such as conversion to Islam (the testament of faith), and the contracting of marriage can legitimately be performed online. However, even within this tradition, the physical presence of an Imam (prayer leader) and fellow congregants are central to legitimizing congregational prayer. For example, following a live screen or radio broadcast of the Imam is not legitimate. Regardless of how immersive, ecologically valid and socially engaging the metaverse becomes, it is hard to see how this advancement might disrupt religious traditions, especially those where physical presence has been a requirement of legitimacy for Millennia.

Furthermore, the financial incentives (cost saving/greater profitability) and safety arguments that drive change in other societal domains will hold less sway in the context of organized religion. A key barrier to a complete “metachurch,” at least within certain traditions, is the dogma/doctrine of physical presence. While some religious organizations might relax their requirements around physical presence and fully embrace the idea of virtual worship,^40^ it will take a theological/jurisprudential rather than a technological shift in other contexts. Within such metaverse-resistant traditions, the associated digital technologies will be limited to, for example, faith-related education, proselytization, pastoral work, and social functions.

## Conclusions

Technological modernization and religion can co-exist and mutually reinforce one another.^51^ Research exploring online religious practice, almost unanimously, suggests that online offerings are more accessible, attracting new participants from traditionally underrepresented demographic groups. The metaverse, with its promise of ecological realism and social presence, might enhance estimations of authenticity in online religious practice. It is possible that rather than distancing or distracting people from religion, future online technology (the metaverse) might make it more accessible and desirable. However, within some religious traditions, certain aspects of religious life (e.g., congregational prayer) are resistant to virtualization/digitalization on doctrinal and jurisprudential grounds. Such sacred functions and associated spaces may represent the last bastions of offline social interaction.

Religion is an essential aspect of social identity and a cornerstone for psychological wellbeing for billions of people worldwide. Further research into online religious participation's health and wellbeing benefits is merited. The metaverse will represent a new way of connecting with this ancient source of human wellbeing.
